# The Influence of Winter and Spring Large-Scale Climate Patterns on the Timing of Male and Female Blackcap Spring Passage at the Baltic Coast

**DOI:** 10.3390/biology15171518

**Published:** 2026-09-03

**Authors:** Agata Pinszke, Magdalena Remisiewicz

**Affiliations:** Bird Migration Research Station, Faculty of Biology, University of Gdańsk, Wita Stwosza 59, 80-308 Gdańsk, Poland; agata.pinszke@phdstud.ug.edu.pl

**Keywords:** spring phenology, protandry, sex-specific migration, climate change, bird migration

## Abstract

Sex-specific adjustments of bird migration phenology to climate change are understudied. We analysed blackcap migration timing using spring (26 March–16 May) ringing data from Bukowo–Kopań (N Poland) in 1982–2025. We assessed the influence of large-scale climate indices in winter (December–February) and spring (March–May) at the beginning (p10), median (p50), and end (p90) of passage of each sex, using multiple regression. Protandry (earlier arrival of males) at the beginning of passage averaged 2.2 days and remained stable over 44 years, because p10 dates advanced by 9 days in both sexes. This aligns with 22-year studies from northern Europe, and with the mating opportunity hypothesis. The median (p50) of passage advanced by 7–9 days, but p90 showed no trends during 1982–2025. Positive winter and spring North Atlantic Oscillation (NAO) and spring Scandinavian Pattern (SCI) were associated with early p10 and p50, and positive winter Indian Ocean Dipole (IOD) with delayed p90, in both sexes alike. These similar responses likely occur because these climate patterns affect broad non-breeding areas of both sexes. We suggest that female blackcaps are as adaptive as males, and advanced migration maximises reproductive success and the chance of second broods in both sexes.

## Simple Summary

Climate change shifts the timing of bird migration, yet whether these environmental pressures affect males and females differently remains poorly understood. We investigated how large-scale climate indices: the North Atlantic Oscillation (NAO), Scandinavian Pattern (SCI), and Indian Ocean Dipole (IOD), which shape weather conditions across Europe and Africa, influence the spring passage of Eurasian blackcaps at the Baltic coast of Poland. Analyses of data from daily standardised mist netting during spring migration (26 March–16 May) at Bukowo–Kopań bird ringing station showed that both sexes have advanced the early and median phases of passage by 7–9 days during 1982–2025. Because both sexes shifted their timing at the same rate, the degree of protandry has remained stable over these 44 years, with males migrating about two days earlier than females. Males and females showed uniform responses to the large-scale climate indices, and migrated later after dry winters in Africa, but earlier after warm winters and springs in Europe. Understanding the extent of adaptability within a migratory species helps to predict how their demography would respond to climate change at different locations, and to inform better international conservation strategies.

## 1. Introduction

Spring migration of birds within the Palearctic–African migration system is an evolutionary adaptation that enables them to benefit from the abundance of food and breeding sites in spring and summer at northern latitudes, where harsh winter limits their year-round residence [1,2,3,4,5]. To maximise individual fitness, migrants time their migration to match local food availability, such as spring vegetation growth and the peak of insects, at their spring destinations [3,6]. However, the optimal timing of spring migration might differ between the sexes, because of different selection pressures on males and females [1,7,8,9,10,11,12]. Climate change in the Northern Hemisphere affects the timing of bird migrations [13,14,15,16,17,18]. However, most studies have focused on the interspecific differences in phenological shifts, while intraspecific variation, including possible sex differences, in responses to climate change remains poorly understood [19].

In many migrant passerines, protandry, which is an earlier arrival of males than females at the breeding grounds, manifests at stopover sites during spring migration [8,9,10,12,20,21]. Changing environmental conditions might influence the degree of protandry (difference in arrival dates of males and females), modifying the costs and benefits of early arrival for each sex [2,10,22,23,24]. Although protandry is common among migrants, differences in arrival times between the sexes are shaped by the selective pressures of a species’ mating system [6,11]. The main mechanism explaining protandry is the mating opportunity hypothesis, where earlier arrival allows males to occupy high-quality territories, increasing their chances of attracting females and improving their reproductive success [7,10,25,26]. Evolutionary models support this hypothesis, suggesting that selection for the timing of male and female arrivals is the main driver of asynchronous sex-specific arrival times [6,21,27]. Differences in arrival timing can also be explained by energy management of each sex. Females in spring should maintain sufficient reserves to meet high nutritional demands of egg production, which often cannot be provided by local foraging [5]. Arriving too early under harsh spring conditions could deplete these reserves, leading to smaller clutches or lower-quality eggs [5,10]. In contrast, sperm production by males is far less energetically demanding, allowing them to take the risk of arriving early [5,11]. The evolutionary advantages of securing the best territories and mates outweigh the potential costs of adverse weather, especially during warmer springs [7,8,10]. This leads to sex-specific migration strategies, in which males prioritise speed of arrival (time-minimising), while females optimise energy costs (energy-minimising) to maintain sufficient body condition [5,11].

The earlier arrival of males at stopover and breeding sites may also result from spatial sex segregation during the non-breeding season, when males and females use separate wintering grounds or different migration routes [6,11,12,28,29,30,31,32]. However, studies using geolocators show that even when the sexes overwinter in the same regions, protandry is driven by behavioural differences [11,33]. In particular, males often depart from their wintering grounds earlier and stay ahead of females throughout the migration [6,33]. Furthermore, migration speed may be sex-specific, as in some species males migrate faster due to a higher rate of energy replenishment or morphological adaptations, such as more pointed wings [5,11].

Over the past few decades, the effects of climate change have become increasingly evident in the Northern Hemisphere, leading to long-term shifts in temperatures and weather patterns [13,15,34]. These climatic changes can directly influence the timing of spring migration [35] and potentially alter the degree of protandry by modifying the costs and benefits of early arrival for each sex [2,22,23,24]. The timing of migration is influenced by environmental factors, such as food abundance or weather conditions at subsequent locations visited by migrants [17,18,22,36,37]. Large-scale climate indices, which are differences in air pressure between distant locations, such as the North Atlantic Oscillation (NAO), Mediterranean Oscillation Index (MOI), and Scandinavian Index (SCAND, hereafter: SCI), or in sea surface temperatures, such as the Indian Ocean Dipole (IOD), reflect atmospheric circulation that shapes winds, temperatures and precipitation over Europe and Africa [17,18,38,39,40,41]. These climate patterns influence the timing of spring migration in many migrant bird species in Europe [17,18,38,39,40,41]. High winter and spring NAO, which bring warm winters and springs to Europe, are associated with early spring arrivals of both short- and long-distance migrants across the continent in such years. The spring phenology of the first group shows a stronger relationship to NAO, attributed to its direct influence on conditions at their wintering and stopover sites in Europe [38,42,43,44,45,46]. However, the arrival timing of long-distance migrants, including the Eurasian blackcap (*Sylvia atricapilla*) (thereafter: blackcap), in southern Europe is also related to winter NAO, likely in response to its influence on conditions across Africa [47]. At our study site in central Europe, the timing of the overall spring passage of six species of long-distance migrants, including blackcap (both sexes jointly), was early with positive winter or spring NAO, and was also related to spring SCI and winter IOD [17]. Recently, spring phenology of the Eurasian wren (*Troglodytes troglodytes*) at our study site was linked to annual variation in winter and spring MOI [18]. These studies guided our choice of climate indices to use as proxies for environmental conditions that blackcaps encounter across their wintering grounds and spring migration routes between Africa and Europe.

Among studies on shifts in avian migration phenology, the possibility of different responses of males and females to changes in climatic conditions has attracted some attention. Earlier analyses of species exhibiting sexual dimorphism showed that in short-distance migrants such as the goldcrest (*Regulus regulus*), the blackbird (*Turdus merula*) and the reed bunting (*Schoeniclus schoeniclus*), the first and middle phases of passage advanced in both sexes between 1976 and 1997 [10]. In long-distance migrants, including the redstart (*Phoenicurus phoenicurus*), the whinchat (*Saxicola rubetra*), the red-backed shrike (*Lanius collurio*), the pied flycatcher (*Ficedula hypoleuca*), and the blackcap, the shifts were less pronounced, and all phases of spring migration advanced in both sexes at a similar rate, thus their degree of protandry remained unchanged over 1976–1997 [10]. However, more recent studies show that, with progressively warmer winters and springs, males begin their migration much earlier than females, leading to an increasing difference in arrival dates, especially in long-distance migrants, such as the pied flycatcher [42,43], the barn swallow (*Hirundo rustica*) [44], the willow warbler (*Phylloscopus trochilus*) [48], the chiffchaff (*Phylloscopus collybita*) [49], and a medium-distance migrant—the song thrush (*Turdus philomelos*) [45]. Uneven shifts in migration timing might result from different responsiveness of the sexes to the same conditions, or from slightly different environmental conditions they experience at non-breeding grounds due to spatial or temporal segregation, or from both mechanisms. The consequent carry-over effects can modify reproductive success, with long-term implications for local population dynamics [12,50].

The Eurasian blackcap, a common migrant passerine with prominent sexual dimorphism, shows remarkable flexibility in adjusting migrations to climate change. This plasticity manifests in shorter migration distances and the establishment of new wintering grounds in the British Isles over recent decades [51,52,53,54]. Additionally, it has been suggested that migrations of the eastern European populations have recently shifted from their traditional wintering grounds in the eastern Mediterranean to more western parts of their wintering range [55]. In earlier studies on the relationships between spring migration phenology of blackcaps and climate indices, the sexes were treated jointly, so potential sex differences have not been considered [17,41]. For these reasons, we selected the blackcap to investigate hypotheses of a potential increase in protandry and sex-specific responses to climate change, similar to those found in other long-distance migratory passerines.

Earlier studies based on about 20 years of data from the late 20th century found no evidence of increasing protandry in the blackcap [10]. Using twice as long a dataset on its spring migration, we set out to reassess this pattern. Firstly, we set out to identify any long-term trends in the spring migration phenology of male and female blackcaps, using a 44-year data series (1982–2025) from the southern Baltic coast. By comparing these trends between the sexes, we aimed to test if the degree of protandry has changed over this period. We expected an increase in protandry over a longer period, considering more rapid climate change and temperature increases across Europe in recent decades. Secondly, we aimed to determine relationships between spring migration timing and large-scale climate indices for male and female blackcaps, and to test if the sexes differ in their relationships to any climate indices. We expected such differences, bearing in mind blackcaps’ protandry and intraspecific differences in sensitivity to environmental conditions in other migrants.

## 2. Materials and Methods

### 2.1. Study Species

**Figure 1:**
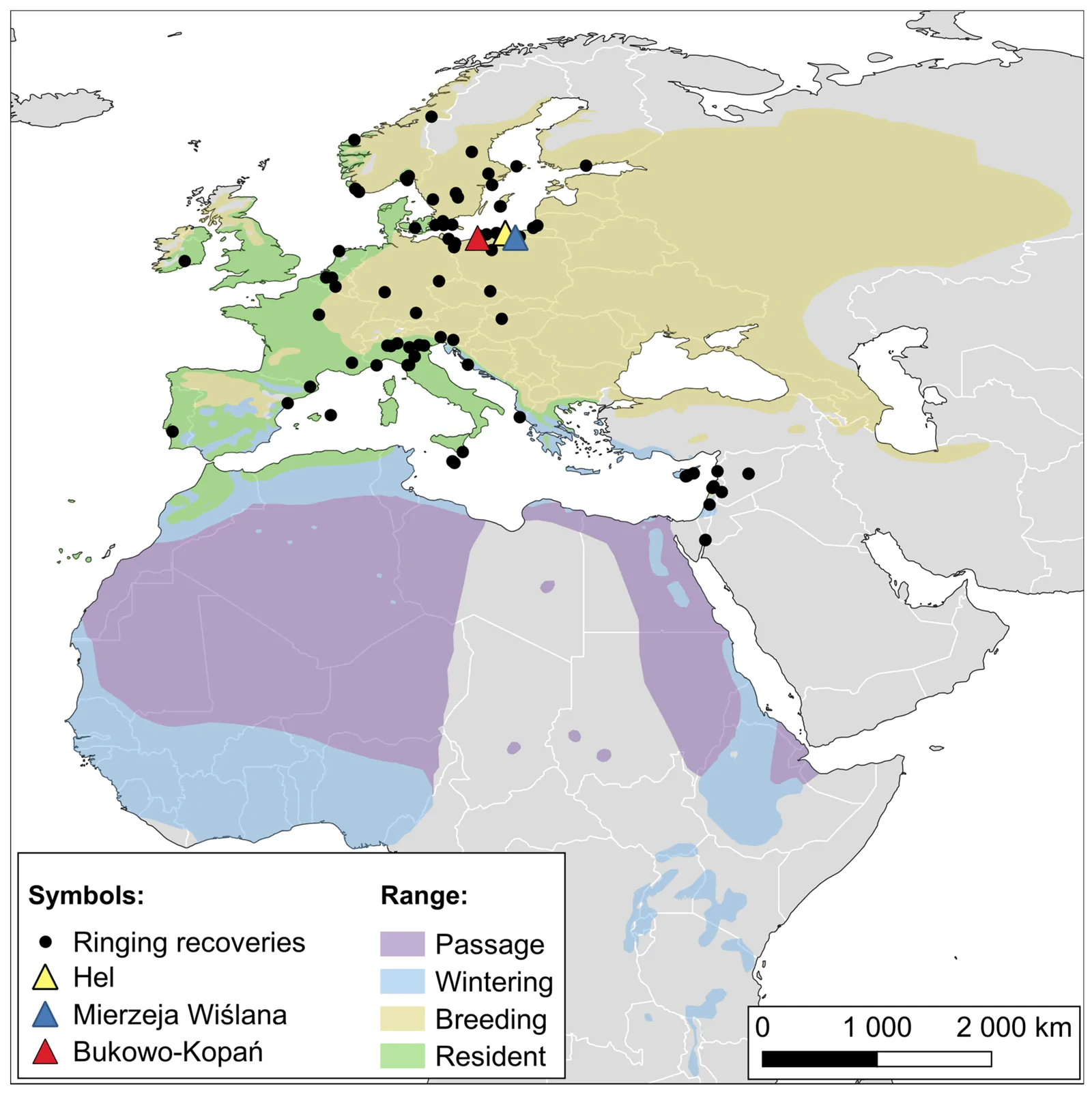
Spatial context of the study. The map shows the locations of the Bukowo–Kopań ringing station (54°19′38″–54°27′54″ N, 16°13′33″–16°25′15″ E) and two other stations of the Operation Baltic Project: Mierzeja Wiślana (54°21′57″ N, 19°23′30″ E) and Hel (54°44′29″ N, 18°33′40″ E), and ringing recoveries of Eurasian blackcaps ringed or recovered at these stations during September 1960–June 2025, against the species’ range [57].

The Eurasian blackcap is a small passerine easily sexed by plumage and size: females have a brown crown, and males are slightly larger with a characteristic black cap [56]. Blackcaps commonly breed across Europe (Figure 1) in habitats with dense undergrowth, such as parks, gardens, and deciduous or mixed woodlands [56].

They are mainly insectivorous, but their pre-migratory fattening is supported by a highly flexible diet including fruits; during periods of invertebrate scarcity they can switch to pollen, nectar, and fruit [56,58]. Blackcaps exhibit considerable variation in migration habits, ranging from completely resident populations breeding in the south and west of Europe (Figure 1) to obligatory long-distance, trans-Saharan migrants in the north and east of the breeding range [56,59]. Central Europe features a migratory divide separating southwest-bound and southeast-bound autumn migration routes, with intermediate populations moving south [60,61]. Additionally, a recent evolutionary shift has led some birds breeding in central and northwestern Europe to winter in the British Isles rather than in their traditional southern wintering grounds [51,52,53,60,62]. The eastern European populations have recently shown some shifts from their historical wintering grounds in the eastern Mediterranean region to more western areas of Europe, closer to the breeding grounds [55]. Populations breeding in western Europe are only partially migratory [56,62].

Blackcaps passing through the southern Baltic coast, which are the focus of this study, originate primarily from populations breeding in Scandinavia and the Baltic region, including local birds breeding near the study site (Figure 1) [61,63]. These populations overwinter across the Iberian Peninsula, northwest Africa, and the eastern Mediterranean region (particularly the Levant), with some individuals migrating as far as west Africa [60,61,63,64,65]. The annual phenology of these populations includes the spring migration from these wintering quarters to the northern breeding grounds during March to mid-May, the breeding season (May to August, with one or two broods), the autumn migration (mid-August to November), and the wintering period (December to February) [56].

### 2.2. Study Site and Methods of Catching Birds

Fieldwork was conducted at the Bukowo–Kopań ringing station, situated on the Baltic Sea coast of Poland (Figure 1), as a part of the long-term Operation Baltic research project. This coastal area, overgrown by mixed forests with fruit bushes, serves as a stopover site for blackcaps heading in spring to northern and northeastern breeding territories and also supports a local breeding population [60,63]. Following the Operation Baltic standard [66], birds were caught in mist nets daily from dawn until dusk. The number of mist nets remained constant within each spring season, but interannual variation ranged from 35 to 61 nets (Supplement Table S1). Upon capture, each bird received a ring, and birds’ sex and age were recorded before release. The station’s habitats and procedures are described in detail by Remisiewicz and Underhill [17].

Our analysis covers spring migration data collected between 26 March and 16 May 1982–2025. Fieldwork was suspended during the springs of 2011 and 2020 due to logistical constraints and the COVID-19 pandemic, resulting in 42 active seasons. Blackcaps caught after 16 May were mostly local breeding individuals, confirmed by their high recapture rates and the presence of brood patches.

To identify geographical regions used by the studied blackcap populations at different life stages, we analysed a supplementary dataset of 105 ringing recoveries. This dataset includes birds ringed at three Operation Baltic stations (Figure 1) and subsequently found elsewhere, as well as foreign-ringed individuals controlled at these stations, between September 1960 and June 2025. We mapped these ringing recoveries (Figure 1) against the species’ geographical distribution [57] using QGIS v. 3.44.5 [67].

### 2.3. Large-Scale Climate Indices

We analysed seven large-scale climate indices that represent climatic conditions across the non-breeding areas and spring migration routes of the blackcap populations we studied. To match these indices to the species’ annual cycle, we calculated seasonal means from monthly values of these indices for two periods: the wintering period (December–February) and spring migration (March–May). These large-scale indices serve as proxies for ecological conditions experienced by blackcaps during these life stages. We provide their brief descriptions below, focused on their positive phases; the negative phase usually brings opposite regional climate effects, but we specify when this pattern occasionally differs.

#### 2.3.1. Mediterranean Oscillation Index (MOI)

**Figure 2:**
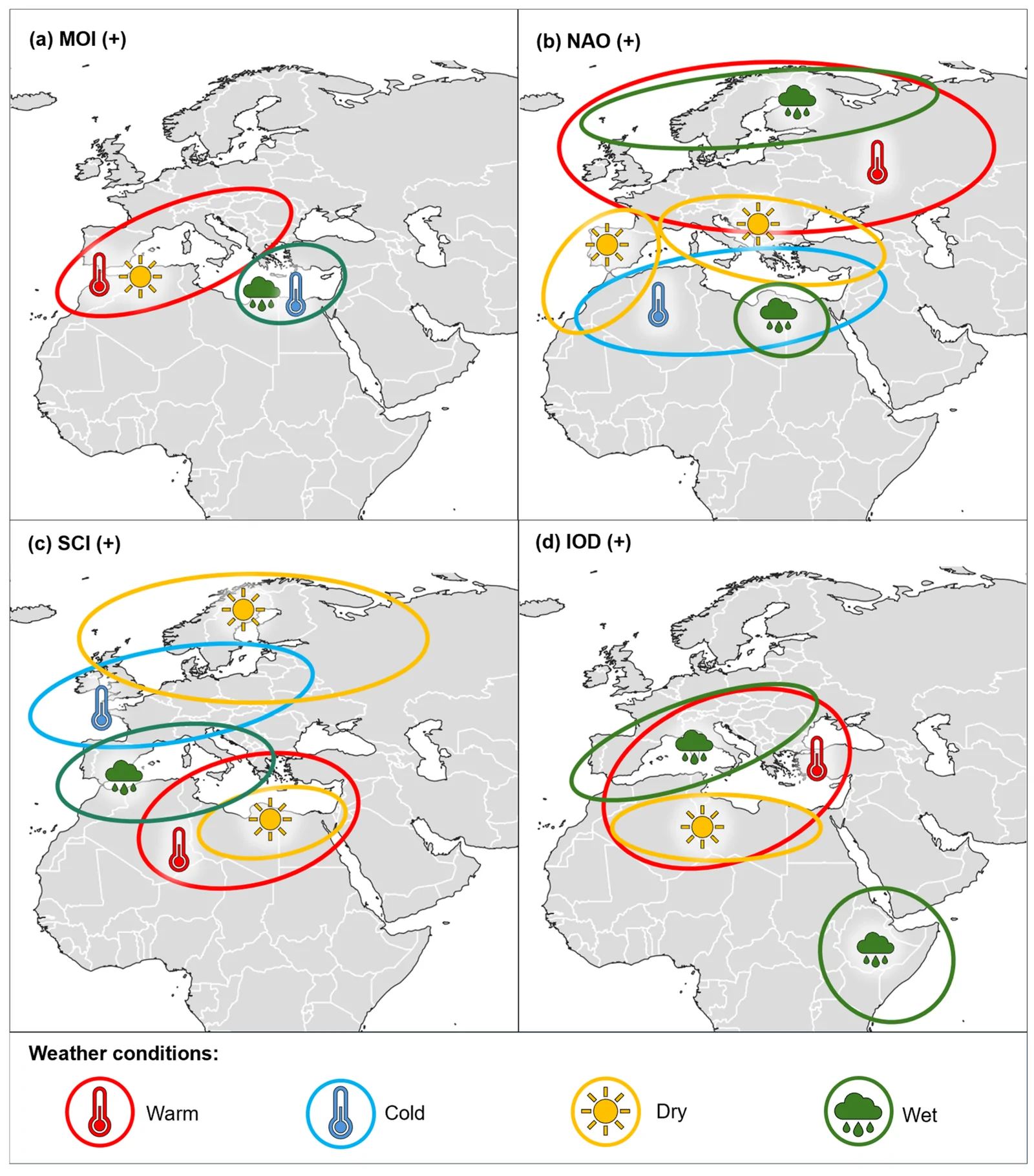
Spatial extent of typical weather conditions associated with the positive phase of the analysed large-scale climate indices in winter and spring (December–May). (**a**) Weather conditions associated with the positive phase of the Mediterranean Oscillation (MOI); (**b**) weather conditions associated with the positive winter and spring North Atlantic Oscillation (NAO); (**c**) weather conditions associated with the positive winter and spring Scandinavia pattern (SCI); (**d**) weather conditions associated with the positive winter and spring Indian Ocean Dipole (IOD).

The Mediterranean Oscillation Index we used in this study is the difference in air pressure between Algiers and Cairo [68,69,70]. The positive winter MOI brings stable, dry, and warm weather across the western and central Mediterranean basin to the Balkan Peninsula, but opposite conditions in the easternmost Mediterranean (Figure 2a) [71,72,73,74,75].

The positive spring MOI maintains dry conditions in the central Mediterranean [76], but with regional exceptions, such as increased precipitation in April and May in Morocco [72], and brings cooler or humid conditions in the Balkan Peninsula [73]. The negative MOI in winter and spring increases precipitation across the western and central Mediterranean [71,74], promoting vegetation and increasing primary production [77,78]. Monthly MOI values were downloaded from the Climatic Research Unit of the University of East Anglia database [79].

#### 2.3.2. North Atlantic Oscillation (NAO)

The North Atlantic Oscillation is the surface sea-level pressure difference between the Azores High and the Icelandic Low [80,81]. The positive winter NAO (Figure 2b) produces strong westerly winds that bring warm, moist winters in northern, central, and eastern Europe. This causes warm winters and significantly reduces the extent, depth, and duration of snow cover across northern, western, and central Europe [82,83]. The northerly airflow exposes southern Europe, the Mediterranean, and North Africa to colder conditions [80,81,84,85,86,87,88]. During positive NAO, winter precipitation increases in northern Europe and the central coast of North Africa, while western and southern Europe, the Balkans, and western North Africa remain drier [80,81,88,89]. In northwestern Africa, this drought can be compounded by dry eastward winds [89], reducing vegetation growth and decreasing primary production [90]. In early spring, the positive NAO maintains these weather patterns [84,91]. Monthly values of NAO index were retrieved from the NOAA Climate Prediction Center database [92].

#### 2.3.3. Scandinavian Pattern Index (SCI)

The Scandinavian Pattern Index is shaped by a pressure difference between Scandinavia and southern Europe (Figure 2c) [93,94,95,96]. The positive winter SCI leads to cold and dry winters across Scandinavia and western, central, and eastern Europe. At the same time, southeastern Europe and the eastern Mediterranean basin experience warm conditions; the whole southern and western Europe, especially the Iberian and Apennine Peninsulas, experience increased rainfall [93,94,97]. The positive spring SCI in spring continues its cooling effect on European weather [98] and maintains dry conditions in the eastern Baltic Sea region [95]. It also generates southerly winds across central and western Europe [99,100] and promotes faster melting of snow cover [94]. During the negative SCI phase, the spatial influence remains identical to the positive phase, but the temperature and precipitation effects are opposite [94,95,97,98]. Monthly SCI values were retrieved from the NOAA Climate Prediction Center database [101].

#### 2.3.4. Indian Ocean Dipole (IOD)

The Indian Ocean Dipole reflects the difference in ocean surface temperatures between the western and southeastern tropical Indian Ocean, which affects the climate in this region directly and the weather patterns in Europe indirectly (Figure 2d) [102]. The positive winter IOD brings above-average rainfall, which increases primary productivity and vegetation growth, in the Horn of Africa and East Africa, but extremely high IOD may cause torrential rains and floods there, which spoil maturing crops [102,103,104,105,106]. In contrast, in southeastern Africa, positive IOD causes droughts and reduces plant productivity [102,103,104]. Positive IOD brings warm winters to most of Europe and North Africa, and increases winter precipitation in southern and western Europe, particularly on the Iberian Peninsula and at the western coast of North Africa [107,108]. Drier conditions occur across the rest of North Africa and the eastern Mediterranean basin (Figure 2d) [108]. High spring IOD, especially in May, leads to unusually warm temperatures across Europe [102,109,110], and increases rainfall in the Horn of Africa [111,112]. The negative winter IOD causes drought in this region [102], as well as in southwestern and eastern Europe and the eastern Mediterranean (Figure 2d) [107,108]. It also brings cold winters to the western and central Mediterranean region and north Africa, but warm conditions in northern and eastern Europe [107,108]. Monthly values of IOD were retrieved from the NOAA international ocean monitoring datasets [113].

#### 2.3.5. Long-Term Trends of Large-Scale Climate Indices and Their Correlations

We checked for multi-year trends in all large-scale climate indices over the study period using simple linear regression models. The spring SCI had the only statistically significant trend and decreased over 1982–2025 (Figure S1). We also checked if these large-scale climate indices were correlated using Pearson’s correlation coefficient, applying Benjamini and Hochberg’s [114] correction for multiple comparisons. Only three correlations were statistically significant (Table 1), and the strongest correlation between the winter NAO and winter MOI was r = 0.68, thus we did not include these two variables in one multiple regression model to reduce multicollinearity.

All correlation coefficients were lower than the recommended threshold of |r| = 0.7, indicating that the effects of multicollinearity on the results of models including these climate variables would be negligible [115].

### 2.4. Methods of Data Analysis

#### 2.4.1. Analysis of Long-Term Trends in Spring Migration Phenology and Protandry

We used only the data from the initial capture for each bird within a season and excluded any unsexed individuals from analyses. We combined all age classes for analyses (adults, second-calendar-year birds, and full-grown), because if the birds from the last, less accurate age category were excluded, the sample sizes in the age/sex groups, especially of adult females, were too small to analyse in one third of the years. Thus, we combined all the age groups, assuming that all blackcaps caught during spring were migrating to their breeding grounds. However, we analysed males and females separately, as this study focuses on sex differences in spring phenology.

First, we used individual capture data to calculate the dates at which 10%, 50% and 90% of all males or females were ringed in each year (Figure S2). We used these dates for each sex, expressed as the day number (1 January = Day 1), as parameters reflecting the timing of three phases of migration: the date of the 10th percentile (p10) as the beginning, the 50th percentile as the middle, and the 90th percentile (p90) as the end phase of passage, as in other studies [10,16,116,117,118,119]. We excluded four years from the analyses when fewer than 5 females were captured, as females were fewer than males among captures (Table S1). Thus, we finally analysed data from 38 springs during 1982–2025, including 2912 females and 4345 males, in total 7257 blackcaps (Table S1).

To estimate multi-year trends in migration timing for each sex, we used linear regression of the dates of p10, p50 and p90 for each sex against the year number from 1982 (YearN), used as a continuous explanatory variable. Our selection of the linear regression approach, used in other studies [10,16,116,117,118,119], was supported by an initial data examination using General Additive Models (GAMs), which showed that multi-year trends of all these dates were nearly linear. We considered the degree of protandry in subsequent phases of passage as the difference in the dates of these percentiles between males and females, as in Tøttrup and Thorup [10]. To check for protandry and to test whether its degree had changed over the years, we ran three multiple linear regression models, one for each of the time series of these percentiles’ dates, including the standardised year (YearSt) and sex (SexN, coded as 0 = males, 1 = females), and their interaction as explanatory variables. We applied the “all subsets” approach using the “dredge” function in the “MuMIn” package [120], and we ranked the models using the Akaike Information Criterion corrected for small sample sizes (AICc). The model with the lowest AICc was considered the best model, which we interpreted.

#### 2.4.2. Estimating the Effects of Large-Scale Climate Indices on Different Phases of Blackcap Spring Migration

To test the effects of large-scale climate indices on the timing of these three phases of passage, we ran multiple linear regression models where the time series of the dates of p10, p50 and p90 in 1982–2025 were response variables, and the explanatory variables included the year, the four large-scale climate indices (NAO, MOI, SCI, IOD) in winter and spring, and the interactions of these climate variables with sex. We selected the multiple regression approach as in other studies [10,17,121] to enable comparisons of results. We used the year as the continuous explanatory variable, not as a random effect, to account for long-term trends in the dataset [16,17,40,122]. All explanatory variables were standardised before being included in the models, because they had different scales. Because these variables were too many to include in one model, we first ran the models separately using the indices for winter months (December–February) in the “winter models” and the indices for spring months (March–May) in separate “spring models”. Because winter MOI and winter NAO were correlated at r = 0.68 (Table 1), to avoid multicollinearity, we ran the partial winter models for two sets of variables: one excluding winter MOI, and the other excluding winter NAO. Thus, we ran nine models: two “winter” models and one “spring” model for each of three migration phases. We conducted the model selection using the “all subsets” approach and model ranking according to AICc, using the “MuMIn” package [120], as previously described.

Comparing these partial “winter” models, we found that models including winter NAO consistently explained a higher proportion of the variation in the migration timing, as they had higher Adjusted R^2^ (Adj R^2^) values than the models including winter MOI. Therefore, in the second step, we used the variables selected in the best “winter” models including winter NAO (not winter MOI), and in the best “spring” models.

Next, for each migration phase (p10, p50, and p90), we ran “winter and spring” models combining winter and spring climate indices selected by the best “winter” and “spring” models. In these multiple regression models, we included those interactions of these indices with sex that were retained by the respective “winter” or “spring” best models in the first step. We applied the same “all subsets” approach and AICc model ranking as earlier, to identify the final best “winter and spring” models. To determine the percentage of explained variation in the response variable, we extracted Adj R^2^ for each final model using the “broom” package [123].

We evaluated multicollinearity in these models using the Variance Inflation Factor (VIF) from the “car” package [124]. In all models, VIF < 3, which indicated no harmful effects of multicollinearity on the results [125]. To test for potential overfitting, we cross-validated the final best models using a three-step approach recommended by [122]: (1) visual inspection of diagnostic plots to identify outliers and highly influential data points based on Cook’s distance; (2) evaluation of the ratio of Adj R^2^ to the multiple coefficient of determination (R^2^), where a ratio approaching 1 indicates an optimal model fit; and (3) comparison of Adj R^2^ with the Predictive R^2^ (predR^2^). The predR^2^ was calculated from the Predicted Residual Error Sum of Squares (PRESS) using a Leave-One-Out Cross-Validation procedure using custom R functions [126]. In this approach, each data point is sequentially removed, the model is refitted, and its ability to predict the omitted observation is evaluated. A minimal difference between Adj R^2^ and predR^2^ indicates that the model is stable and not overfitted [126]. Additionally, we evaluate the presence of first-order temporal autocorrelation in the model residuals, using the Durbin–Watson test. This application of multiple regression, evaluation of final models’ multicollinearity and stability followed earlier studies [18,40,41]. The final “winter and spring” models provided estimates of the effect of each selected climate variable on the dates of p10, p50, and p90 of the spring passage. We visualised these effects as scatter plots showing relationships of these variables to the dates of these phases of passage and fitted linear regression lines, using the “ggplot2” package [127]. All statistical analyses were conducted in R 4.4.2 [128].

## 3. Results

### 3.1. Protandry and Sex-Specific Long-Term Trends in the Timing of Spring Migration of Blackcaps

**Figure 3:**
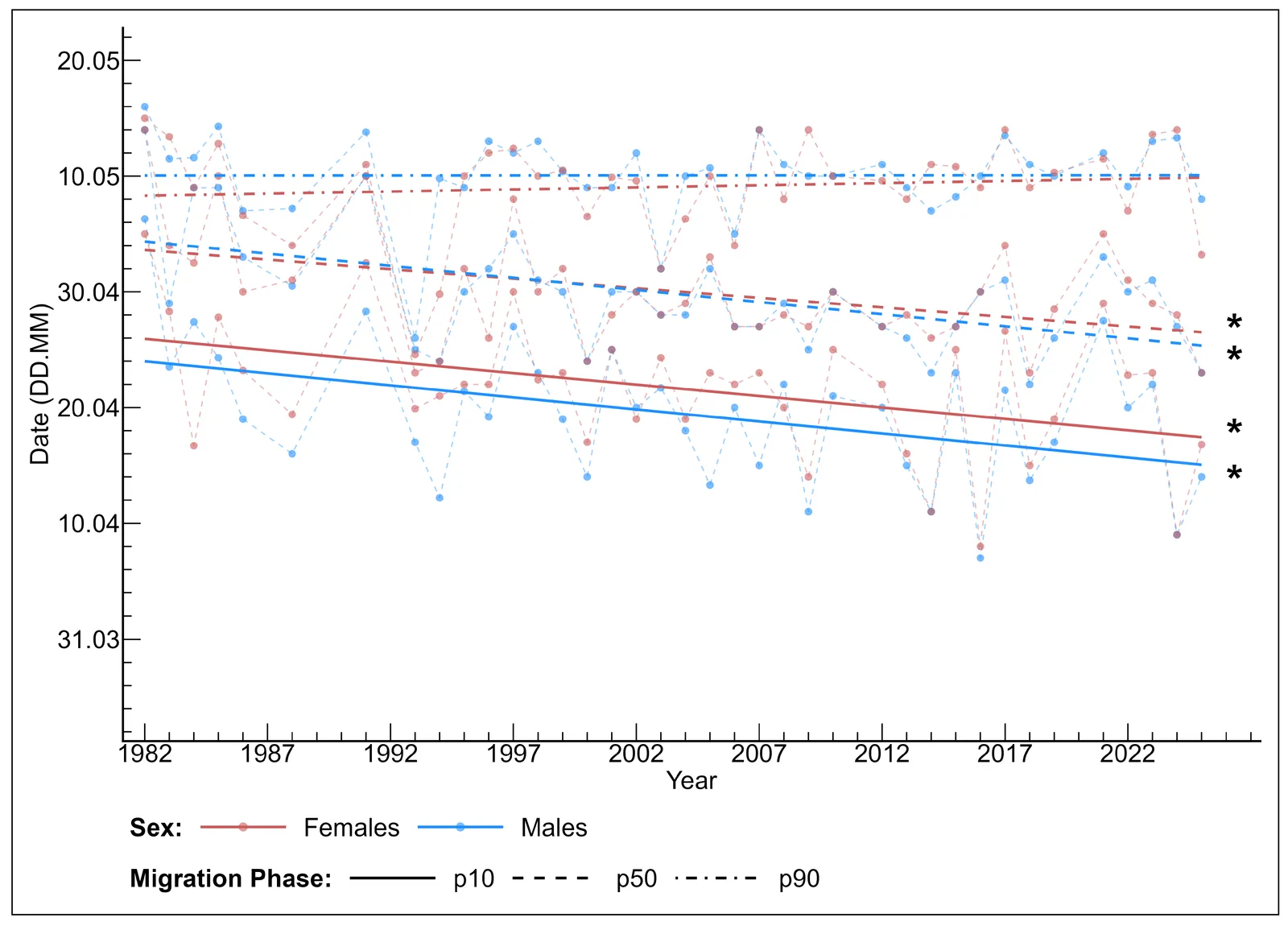
Multi-year trends in the timing of the first (p10), median (p50), and last (p90) phases of spring migration for male and female Eurasian blackcaps at Bukowo–Kopań in 1982–2025. Circles represent the dates of the respective percentiles of passage in each year plotted against the year, for each sex separately. Lines show fitted linear regression trends; asterisks (*) indicate statistically significant trends. The details of the regression are in Table S2.

During 1982–2025, the dates of the beginning (p10) of spring passage for both sexes shifted earlier by 9 days (Figure 3; Table S2). Protandry in blackcaps at Bukowo–Kopań occurred at the beginning of spring passage because p10 was on average 2.2 days earlier for males than females (Figure 3; Tables S3 and S4). This degree of protandry remained stable over 1982–2025 (Figure 3, Tables S3 and S4), as indicated by the parallel regression lines (Figure 3) and a lack of the YearSt:SexN interaction in the best model for p10 (Table S4). The median dates (p50) of passage did not differ between the sexes (Figure 3), as confirmed by the absence of the sex effect (SexN) in the best model (Table S4). The p50 dates for males shifted earlier by 9 days and for females by 7 days, over 1982–2025 (Figure 3; Table S2). These advancing trends were similar, as indicated by no YearSt:SexN interaction in the best model (Table S4). The dates of the end of migration (p90) showed no trends over time and did not differ between the sexes (Figure 3; Table S3), as shown by no effect of sex (SexN) in the best model (Table S4).

### 3.2. Effects of Large-Scale Climate Indices on Spring Migration Timing

The “winter” models including NAO (but not MOI) explained 7–27% of the variation in the timing of different phases of spring migration (Table S6), while the models including MOI (but not NAO) explained 7–25% of this variation (Table S8). Because the “winter” models including winter NAO consistently explained a higher proportion of the variation than those including MOI (Tables S6 and S8), we used the outcome from the first approach in the combined “winter and spring” models. The best “spring” models explained from 0% to 44% of the variation in the timing of p90 and p10, respectively (Table S10).

**Figure 4:**
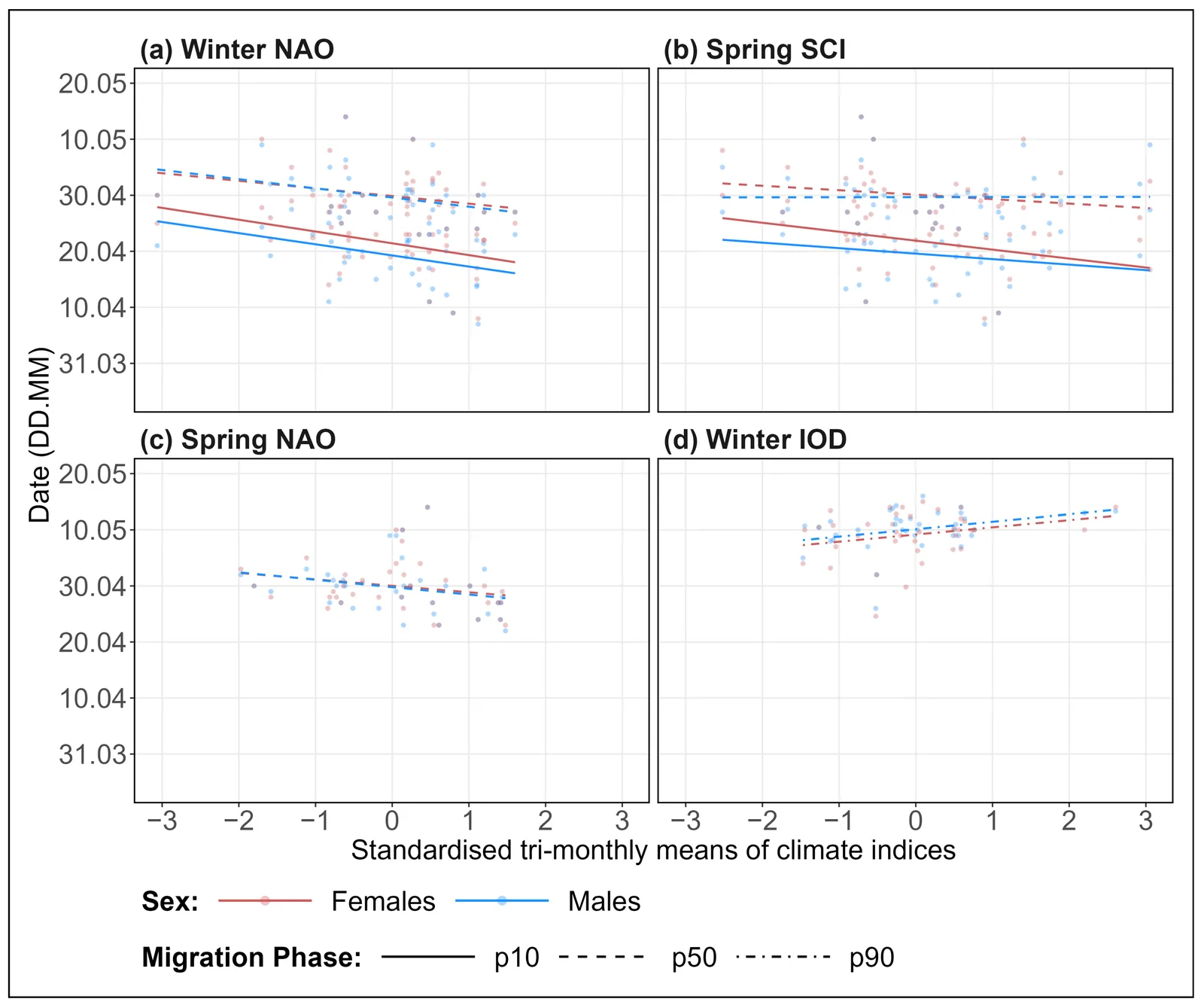
Relationships between the timing of the selected percentiles (p10, p50, p90) of spring migration for male and female Eurasian blackcaps at Bukowo–Kopań in 1982–2025 and the large-scale climate indices selected in the best “winter and spring” models (Table S13). Points represent the dates of the respective percentiles of passage plotted against the values of the climate index in a given year. Linear regression lines show the modelled trends. (**a**) The effect of the winter NAO on the first (p10) and median (p50) phases of passage; (**b**) the effect of the spring SCI on the first (p10) and median (p50) phases of passage; (**c**) the effect of the spring NAO on the median (p50) phase of passage; and (**d**) the effect of the winter IOD on the end (p90) phase of passage.

The best “winter and spring” model (Table S11) explained 48% of the variation in the date of the beginning of passage (p10) at Bukowo–Kopań during 1982–2025 (Table S12). We found no differences between the sexes in the relationships of p10 to the climate indices. For both sexes, p10 was early with high winter NAO (Figure 4a) and high spring SCI (Figure 4b), or late with low values of these indices, as these relationships work both ways (Tables S12 and S13).

For the median date of passage (p50), the best model explained 39% of its variation in 1982–2025 (Table S12). No sex-specific differences occurred in the effects of large-scale climate indices. The median for both sexes was early with high winter NAO (Figure 4a, Table S13), high spring NAO (Figure 4c, Table S13), and high spring SCI (Figure 4b, Table S13). The best model for the end of passage (p90) explained 7% of its variation over the 44 years (Table S12). The sexes did not differ in their relationships to any of the climate indices. The date of p90 for both sexes was late with high winter IOD, or early with low IOD (Figure 4d; Tables S12 and S13).

Diagnostic tests confirmed the stability of the final “winter and spring” models (Table S14, Figure S3). Minimum discrepancies between Adj R^2^ and pred R^2^ and high ratios of Adj R^2^ to R^2^ (range: 0.84–0.94) indicated that the models were not overfitted (Table S14). Visual inspection of diagnostic plots confirmed that the assumptions of the regression models were met, with no highly influential outliers (Figure S3). The Durbin–Watson test indicated positive autocorrelation in the residuals (DW statistics: 0.91–1.25, Table S14). Such autocorrelation is common in multi-decadal ecological time series, as successive years often share similar environmental conditions [129,130]. However, given the high stability of our models, this did not affect the reliability of the identified climatic relationships (Table S14).

## 4. Discussion

Our study shows that both male and female blackcaps advanced their spring passage through the southern Baltic coast over 1982–2025 at similar rates. The first phase of passage (p10) shifted earlier by 9 days in both sexes, thus protandry remained stable over these 44 years, opposite to what we expected, considering the changes in protandry in other passerines [45,48,131]. The two-day protandry, the most apparent at the first phase, decreased at the subsequent phases of blackcaps’ passage, although the median phase advanced for males and females by 9 and 7 days, respectively. Contrary to our expectations, annual variation in migration timing was related to large-scale climate indices in both sexes similarly, but the timing of subsequent phases of passage in both sexes was shaped by different climate indices. The first (p10) and median (p50) phases of passage were early with positive winter and spring NAO, and spring SCI, but the last phase (p90) was delayed after positive winter IOD, in males and females alike, suggesting that successive cohorts of both sexes arrived along different migration routes. We discuss these findings in the context of the mating opportunity hypothesis and changing climate conditions across blackcap wintering grounds and migration routes.

### 4.1. Long-Term Phenological Trends in Both Sexes and Drivers of Spring Protandry

Protandry is common among migratory birds, driven mainly by the selective pressures of the mating system [6,11]. The mating opportunity hypothesis explains that earlier arrival enables males to occupy high-quality territories to improve reproductive success [10,26]. These phenological differences are related to energy management. Females must maintain sufficient fat and protein reserves for egg production, so they optimise energy costs and avoid harsh conditions early in spring that could deplete these essential reserves [5]. In contrast, males prioritise migration speed over energy saving [11]. Thus, blackcap males exhibit a time-minimising strategy and females an energy-maximising strategy during spring migration [132]. Furthermore, the earlier passage of males may result from spatial sex segregation at the non-breeding grounds. In blackcaps, males might winter in greater proportion at more northern latitudes than females, having a shorter migration distance to cross [30,133,134].

We suggest that both mechanisms contribute to the spring protandry we found at the first phase (p10) of passage at Bukowo–Kopań over 1982–2025, in line with earlier studies [10,135]. The protandry among the first migrants supports the mating opportunity hypothesis. Competition for breeding opportunities creates evolutionary pressure on males [7,136], as the first-arriving individuals secure the best territories and attract the earliest females [137,138,139]. In contrast, the timing of the median (p50) and the end (p90) phases of passage at our station was similar for both sexes, suggesting that the pressure for early male arrival fades as the season progresses. The lack of protandry in the median dates of passage contrasts with results from Helgoland (North Sea, Germany) and Christiansø (Baltic Sea, Denmark), where this phase of passage was significantly earlier in males than in females [135]. This discrepancy might result from differences in the composition of populations passing through these stations compared to Bukowo–Kopań, or the different time periods analysed (1982–2025 in our study vs. 1976–1997 in Rainio et al. [135]).

The degree of protandry at the first phase of passage (p10) did not change over our 44-year study period, as males and females advanced their migration timing at a similar rate. Furthermore, the median of passage (p50) advanced similarly for males and females, by 9 and 7 days, respectively, despite no clear protandry at that phase of migration. This uniform phenological shift aligns with earlier studies, which also reported no changes in the degree of protandry over 1976–1997, both for the combined data from Helgoland and Christiansø [135], and for Christiansø alone [10]. The similar advancements of both sexes suggest that they respond to similar environmental conditions shaped by oscillation patterns over large areas, where the sexes likely occur in a close sequence [134]. Males and females probably also fine-tune the progress of passage across Europe using the same external cues, like temperature or food availability at stopover areas, rather than sex-specific cues [10,140,141]. The parallel phenological shifts suggest that among early arrivals both sexes compete for mates [10] and earlier spring passage benefits both sexes equally. Mild springs might enable males to reach breeding grounds early to maximise mating opportunities, and allow females to lay eggs earlier, extending the breeding season and increasing chances of raising two clutches for both sexes [20,26].

Interestingly, resident birds such as great tit (*Parus major*) and blue tits (*Cyanistes caeruleus*) exhibit similar phenological plasticity, shifting their breeding dates earlier [142,143]. These populations utilise local environmental cues, such as springtime warming and early leafing, to synchronise their reproduction with the accelerated peak of food resources [144].

The advancement of the start of migration by 9 days (for both sexes) and of the median by 7–9 days at Bukowo–Kopań is consistent with the shift to earlier median migration dates during 1976–1997 for combined data from Helgoland and Christiansø [135]. Our results also correspond with the trends observed at Christiansø alone [10] that indicated an advance in arrival at all migration phases (5%, 50%, and 95%); though, these trends were not significant, likely because of a shorter time series than ours. More recently the first and last 5% of blackcaps’ passage significantly advanced during 1984–2022 at the Blåvand bird observatory in Denmark; however, unlike our study, this analysis combined males and females [119]. The lack of any multi-year trend in the timing of p90 in our study differs from the significant trend at Blåvand [119], probably because at Bukowo–Kopań the local breeding blackcaps occurring at the end of spring masked any trends. A weaker phenological shift with the progress of the passage is a common pattern across all the mentioned stations [10,119]. This might result from a slower migration of birds in the “tail” of passage, which often includes weaker individuals, which likely were “outcompeted” by the preceding fitter individuals, but might still be able to raise some chicks [145], hence smaller time pressure. Furthermore, this late phase mainly consists of females and young males [131,146]. These individuals face weaker selection pressure to migrate early because females do not establish territories and young males have lower chances to secure prime breeding spots than adults [7,8,21]. The overall shift to earlier passage for the first half of the population suggests that blackcaps respond to increasingly mild conditions on their wintering grounds and along spring migration routes [2,10,22], which increases their chances to raise two clutches during extended breeding season [26]. While our results highlight the role of large-scale climate patterns, we suggest that these phenological shifts might be co-influenced by other ecological factors. These could include habitat changes along the flyways, fluctuations in stopover site quality, and broader population-level shifts, such as the increasing numbers of blackcaps wintering in western and northern Europe [52,53,54,55]. Similar advancement suggests that earlier spring passage benefits both sexes equally. Mild springs might enable males to reach breeding grounds early to maximise mating opportunities, and allow females to lay eggs earlier, extending the breeding season and increasing chances of raising two clutches for both sexes [20,26].

### 4.2. Similar Responses of Both Sexes to Large-Scale Climate Indices

Our key finding is a lack of differences in male and female blackcap relationships to any of the large-scale climate indices. This differs from our initial expectations that males should respond more strongly to some climate patterns, especially those that shape winter and spring conditions in Europe, as male blackcaps generally winter closer to their breeding grounds than females [6,11,12,28,29,30,31,32]. Unlike the song thrush, in which after warm winters and springs the males arrived in the Baltic region much earlier than the females [45], in the blackcap the relationships of both sexes to climatic conditions were similar, despite the protandry at the first phase of passage (p10) at Bukowo–Kopań. This difference might reflect species-specific phenological plasticity, with the song thrush and both sexes of the blackcap showing the same level of flexibility in response to changing conditions. Both species share part of their wintering grounds in the Mediterranean region, but Redlisiak et al. [45] analysed regional mean temperatures in southern and southwestern Europe, unlike our study. If male and female song thrushes are partly segregated across the wintering grounds, as other passerines, they might be exposed to varied local temperatures, which could affect their migration differently. This contrasts with the large-scale climate factors we analysed for blackcaps, which have a climatic footprint over much larger areas of the blackcap wintering range from northern Africa through the Mediterranean to central Europe [80,147]. Even though females winter farther south than males due to spatial segregation [11,30], both sexes likely experience the same broad weather conditions dictated by these large-scale oscillations and adjust their migration timing accordingly. Additionally, female blackcaps appear to be as phenologically adaptive as males, likely because the benefits of advancing spring migration and increasing the chance for second broods balance the risks posed by cold spells in spring similarly in both sexes [20,26]. This uniform response of males and females is consistent with findings from Helgoland and Christiansø, where the winter NAO did not affect the degree of protandry in blackcaps [135], and with the conclusion that both sexes respond similarly to changing climatic conditions [10]. However, this uniform response is atypical compared to other passerines, for example the barn swallow, the willow warbler, the chiffchaff and the song thrush, which show increasing differences in arrival dates between the sexes with progressively warmer winters and springs [42,43,44,45,48,49]. This suggests that uneven shifts in migration timing are not a common pattern among all migratory birds. In species with remarkable flexibility, such as the blackcap, the benefits of early arrival, such as occupying high-quality territories for males [7,10] and extending the breeding season for females [20,26], likely create similar selective pressures on both sexes, allowing them to adjust to changing environmental conditions and maximise reproductive success. Protandry in blackcaps appears to be maintained by the selective pressures described by the mating opportunity hypothesis [7,10,20], regardless of the weather conditions in a given season.

### 4.3. Effect of the North Atlantic Oscillation (NAO) on Spring Migration Timing

Positive winter NAO was related in both sexes to an advanced first (p10), and, with a weaker effect, to the median (p50) phases of passage, but not to its end phase (p90), a pattern similar to the responses to winter NAO in blackcaps migrating through other locations [10,116,119,135]. Our results for the first phase of passage contrast with no significant relationship between the first arrival dates of blackcaps and winter NAO shown by long-term (1881–2007) observations from the Czech Republic [148]. This suggests that first blackcaps arrive there from areas under a weaker climatic influence of NAO, like the eastern Mediterranean or southeast of Europe [61], than the first migrants at more northern and coastal locations in Europe, including Bukowo–Kopań. The relationship between NAO and the first phase of passage at our station suggests that the first migrants winter close to the breeding grounds, in areas where positive NAO brings mild winters, such as western and central Europe [80,88]. Warm winters reduce energy expenditure on thermoregulation and lead to an earlier availability of insect prey, allowing these short-distance migrants to reach migration condition faster and depart early. The relationship we found between winter NAO and advancement of p50 contrasts with earlier results from Helgoland and Christiansø, where no analogous effect was found [135], possibly because they analysed a shorter time series. The relationship we revealed might also reflect a more recent shift in wintering patterns of the eastern population of blackcap, from eastern to more western wintering grounds [55], which are under climatic influence of NAO. Positive spring NAO was related to the early median phase of passage (p50) for both sexes, in line with earlier results showing such a relationship for both sexes jointly [17]. Positive NAO brings south-westerly winds and warm spring across central and western Europe [80,84,85,91]. This effect of NAO is most pronounced in March, about the time that migrant blackcaps moving northward enter this region. Winds from the southwest can assist migrants in their movement across Europe to the northeast. Warm conditions might benefit migrants more indirectly, accelerating the development of spring vegetation and increasing insect abundance at stopover sites [22,149]. Additionally, the reduced snow cover across western and central Europe [82,83] makes these stopover sites accessible earlier in the spring. All these effects of positive NAO favour quick fuel accumulation along migration routes, allowing birds to maintain their early passage.

### 4.4. Effect of the Scandinavian Pattern Index (SCI) on Spring Migration Timing

Positive spring SCI, which brings warm weather and thus likely favourable food conditions to the central and western Mediterranean [93,94,97], was associated with earlier first (p10) and median (p50) phases of passage of both sexes at Bukowo–Kopań. This suggests the first half of migrants benefit from these conditions, which allow for quick fuel accumulation and thus shorter stopovers during spring migration. Following the mating opportunity hypothesis [7,10,20], we suggest that the migrants that form the first phase of passage (p10) winter close to the breeding grounds, probably at the northern edges of the central and western Mediterranean region. With positive SCI, they might experience the spring peak in food availability in these areas, which enables an early departure for migration. Birds that form the middle phase (p50) of passage, arriving from more southern wintering areas, might experience such conditions during their stopovers in these areas, which results in an analogous phenological shift. Furthermore, the south-westerly and southerly winds across western and central Europe during positive spring SCI can directly assist early migrants in their movement across Europe to the northeast, contributing to the earlier beginning (p10) and median (p50) of passage at our station. This earlier arrival is beneficial, as positive SCI accelerates spring snowmelt across central Europe and Scandinavia [99,150], making subsequent stopover sites and northern breeding grounds accessible earlier in the season. These phase-specific responses align with the previously found relationship between the positive spring SCI and advancement of the overall spring passage of the blackcap at Bukowo–Kopań [17]. By dividing the migration into phases, our study reveals that this overall relationship has been primarily driven by a response of the first half of migrants to environmental conditions shaped by SCI.

### 4.5. Effect of the Indian Ocean Dipole (IOD) on Spring Migration Timing

Positive winter IOD, which brings high rainfall to East Africa [111] but dry spells to the eastern Mediterranean [108,151], was related to the delayed end of migration (p90) in both sexes. This suggests that blackcaps that form the end of spring passage at Bukowo–Kopań arrive from the eastern part of wintering grounds or stopover areas in the eastern Mediterranean region, as indicated by ringing recoveries (Figure 1) [61]. The delayed last phase (p90) of blackcaps’ passage after high winter IOD corresponds to late overall spring passage of the common redstart and the pied flycatcher, and a late end of the chiffchaff’s passage at Bukowo–Kopań with such conditions [17,41]. These delays might be attributed to heavy rains and floods in East Africa with extremely high winter IOD, as in 2019/2020 [111]. Rainfall and temperature conditions in Africa in February–March can affect migrants’ departure from these areas, directly by influencing their energy requirements during fuelling, and indirectly by affecting resource availability and conditions for birds [47,152]. High IOD, related to a lack of rain in the Mediterranean region, limits vegetation growth and food availability [153,154]. Such unfavourable conditions might extend the time the birds need to build fuel reserves and delay their departure from wintering or stopover sites in this area. Analogies between the patterns in blackcap and other species indicate that IOD similarly influences several long-distance migrants, probably those that arrive at the southern Baltic coast from the southeast, along similar spring migration routes. Unfavourable conditions for birds related to extremely high winter IOD in East Africa and the eastern Mediterranean might compound the delay in birds’ migration along this route and ultimately delay their arrival at the Baltic coast.

### 4.6. Study Limitations and Broader Ecological Context

Although our 44-year study provides clear long-term trends, some methodological limitations should be considered while interpreting our results. Furthermore, annual capture numbers of males and females could be influenced by local and interannual changes in habitat and stopover site quality. Capture probabilities might also be affected by minor variations in catching effort; although we aimed for methodological consistency, the exact number of mist nets varied between seasons (Table S1), and the location of mist netting slightly shifted over the 44-years. With the available data, we could not isolate the effects of individual body condition or age on the timing of passage. Our findings, such as the stable degree of protandry and uniform responses to climate indices, align with patterns from nearby Baltic and North Sea stations like Christiansø and Helgoland [10,135], but our data come from a single ringing station. Thus, they represent a location-specific mix of blackcaps arriving there along the western and the eastern flyways, and of local breeding birds and individuals migrating farther north to Scandinavia [60,61,62,63]. Therefore, the phenological patterns we observed might not fully capture the dynamics along all flyways across Europe, and might differ at other locations, thus comparisons with patterns at other stations might lead to further interesting findings.

## 5. Conclusions

We showed that both male and female blackcaps advanced the first half of their spring migration on the Baltic coast at similar rates; thus, the two-day protandry in the first phase of their passage remained stable over the 44 years of our study. This stable protandry can be explained by the mating opportunity hypothesis, which suggests that selective pressures preserve the difference in spring timing between the sexes, regardless of the interannual variation in weather conditions. Despite this protandry, males and females responded uniformly to winter and spring climate patterns, suggesting that these conditions similarly affect broad non-breeding grounds of both sexes. We suggest that females are as phenologically adaptive as males, to maximise a common reproductive outcome for both sexes. Such phenological response to climate change apparently benefits the species, considering its almost threefold increase in numbers across Europe, including the Baltic region, since the 1980s [128].

## Figures and Tables

**Table 1:** Pearson’s correlation coefficients between the winter (December–February; indicated as WIN) and spring (March–May, indicated as SPR) temperature variables in 1982–2025. Bold values indicate statistically significant correlations, after Benjamini–Hochberg correction for multiple comparisons with an accepted 5% level of false positives.

Climate Variable	NAO_SPR	IOD_WIN	IOD_SPR	SCI_WIN	SCI_SPR	MOI_WIN	MOI_SPR
NAO_WIN	0.19	−0.17	0.03	**−0.55**	−0.11	**0.68**	−0.16
NAO_SPR		−0.25	−0.10	**−0.42**	0.06	0.33	−0.02
IOD_WIN			0.37	0.07	0.10	−0.18	0.18
IOD_SPR				0.09	−0.23	−0.16	0.29
SCI_WIN					0.01	**−0.55**	0.14
SCI_SPR						−0.03	−0.32
MOI_WIN							−0.31

## Data Availability

Long-term ringing data are accessible via the Global Biodiversity Information Facility (GBIF) [155].

## Supplementary Materials

The following supporting information can be downloaded at https://www.mdpi.com/article/10.3390/biology15171518/s1, Figure S1: Long-term trends in large-scale climate indices used in the analysis. Statistically significant trends are indicated by an asterisk (*) next to the variable abbreviation; Figure S2: Visual representation of the method of calculating the dates of spring migration phases (p10, p50, and p90) for males and females of Eurasian blackcaps, using the 2009 spring season as an example. The solid stepped lines illustrate the cumulative curve of passage (left y-axis), while the bars show the daily number of captured individuals (right y-axis). The horizontal and vertical lines show how the capture of 10%, 50%, and 90% of all males of females indicated the dates of the respective migration phases. (a) Visual representation of deriving the dates of the three phases of passage for males; (b) visual representation of deriving the dates of the three phases of passage for females; Figure S3: Diagnostic plots for the final best “winter and spring” linear regression models for the dates of the beginning (p10), median (p50), and end (p90) of spring migration. The plots present residuals versus fitted values, normal Q-Q plots, scale-location plots, and residuals versus leverage to assess regression assumptions, including linearity, normality of residuals, and identification of highly influential data points (Cook’s distance); Table S1: Numbers of captured males, females, and all Eurasian blackcaps (*Sylvia atricapilla*) caught, and of the nets used during each spring migration season (26 March–16 May) at Bukowo–Kopań in subsequent years of the period 1982–2025. Grey fields—seasons which were excluded from analyses, because the station did not operate or fewer than five females were caught. Total for analyses (in both font) indicates the numbers of individuals considered in analyses; Table S2: Linear regression parameters for the long-term trends in the dates for the beginning (p10), median (p50), and end (p90) of spring migration of the Eurasian blackcaps at Bukowo–Kopań in 1982–2025 (Figure 3). The table presents the slope coefficient (β), intercept, standard error (SE), and significance level (p). ‘β x years’ is the estimated total shift in arrival timing (in days) during the analysed period, calculated by multiplying the slope coefficient by the number of years (44 years). A negative β value indicates an advance in the date of the analysed percentile over time; Table S3: Model selection for the linear regression models for the dates of the beginning (p10), median (p50), and end (p90) of spring passage, used as response variables. The explanatory variables in the full models included sex (SexN), standardised year (YearSt), and their interaction (YearSt:SexN). The table presents the coefficient estimates for the intercept and the explanatory variables selected in each model, followed by the model statistics: degrees of freedom (df), Akaike Information Criterion corrected for small sample sizes (AICc), the difference in AICc relative to the best model (delta AICc), and the Akaike weight (weight). The table presents the set of models with a delta AICc < 2. Bold digits indicate variables selected in the best model. Parameters of these best models are presented in more detail in Table S4; Table S4: Parameters of the best linear regression models for the dates of the beginning (p10), median (p50), and end (p90) of spring migration used as response variables, and the explanatory variables: standardised year (YearSt), sex (SexN), and their interaction (YearSt:SexN). The best models were selected based on the lowest AICc (Table S4). The table presents the coefficient estimate (Estimate), its standard error (SE), t-value (t), and significance level (p) for the intercept and explanatory variables selected in the best models. The estimates for the YearSt:SexN interaction indicate the change in the degree of protandry over the years; Table S5: Model selection for the “winter” models including NAO (without MOI) of relationships between dates of the beginning (p10), median (p50), and end (p90) of spring migration of the Eurasian blackcap at Bukowo–Kopań and winter large-scale climate indices, excluding the winter MOI variable, in 1982–2025. The candidate explanatory variables are listed in the table heading. Abbreviations of the temperature variables are provided in Table 1. The table presents the coefficient estimates for the intercept and the explanatory variables selected in each model, followed by the model statistics: degrees of freedom (df), Akaike Information Criterion corrected for small sample sizes (AICc), the difference in AICc relative to the best model (delta AICc), and the Akaike weight (weight). The table presents the set of models with delta AICc < 2. Bold digits indicate variables selected for the best partial “winter” model. Parameters of this best model are presented in more detail in Table S6; Table S6: Parameters of the best “winter” linear regression models including NAO (without MOI), for the dates of the beginning (p10), median (p50), and end (p90) of spring migration and winter variables in 1982–2025. The best model had the lowest AICc. The table presents the coefficient estimate (Estimate), its standard error (SE), t-value (t), and significance level (p), and the Variance Inflation Factor (VIF) for the intercept and the explanatory variables selected by the best model, as well as the Adjusted R^2^ (Adj R^2^) for the entire model; Table S7: Model selection for the “winter” models of relationships including MOI (without NAO) between dates of the beginning (p10), median (p50), and end (p90) of spring migration of the Eurasian blackcap at Bukowo–Kopań and winter large-scale climate indices, excluding the winter NAO variable, in 1982–2025. The candidate explanatory variables are listed in the table heading. Abbreviations of the temperature variables are provided in Table 1. The table presents the coefficient estimates for the intercept and the explanatory variables selected in each model, followed by the model statistics: degrees of freedom (df), Akaike Information Criterion corrected for small sample sizes (AICc), the difference in AICc relative to the best model (delta AICc), and the Akaike weight (weight). The table presents the set of models with delta AICc < 2. Bold digits indicate variables selected for the best partial “winter” model. Parameters of this best model are presented in more detail in Table S8; Table S8: Parameters of the best “winter” linear regression models including MOI (without NAO), for the dates of the beginning (p10), median (p50), and end (p90) of spring migration at Bukowo–Kopań in 1982–2025 and winter variables in 1982–2025. The best model had the lowest AICc. The table presents the coefficient estimate (Estimate), its standard error (SE), t-value (t), and significance level (p), and Variance Inflation Factor (VIF) for the intercept and the explanatory variables selected by the best model, as well as the Adjusted R^2^ (Adj R^2^) for the entire model; Table S9: Model selection for the “spring models” of relationships between dates of the beginning (p10), median (p50), and end (p90) of spring migration of the Eurasian blackcap at Bukowo–Kopań and spring large-scale climate indices in 1982–2025. The candidate explanatory variables are listed in the table heading. Abbreviations of the temperature variables are provided in Table 1. The table presents the coefficient estimates for the intercept and the explanatory variables selected in each model, followed by the model statistics: degrees of freedom (df), Akaike Information Criterion corrected for small sample sizes (AICc), the difference in AICc relative to the best model (delta AICc), and the Akaike weight (weight). The table presents the set of models with a delta AICc < 2. Bold digits indicate variables selected for the best “spring” model. Parameters of this best model are presented in more detail in Table S10; Table S10: Parameters of the best “spring” linear regression models for the dates of the beginning (p10), median (p50), and end (p90) of spring migration at Bukowo–Kopań and spring variables, in 1982–2025. The best model had the lowest AICc. The table presents the coefficient estimate (Estimate), its standard error (SE), t-value (t), and significance level (p), and the Variance Inflation Factor (VIF) for the intercept and the explanatory variables selected by the best model, as well as the Adjusted R^2^ (Adj R^2^) for the entire model; Table S11: Model selection for the “winter and spring” models of relationships between dates of the beginning (p10), median (p50), and end (p90) of spring migration of the Eurasian blackcap at Bukowo–Kopań and for combined winter and spring temperature variables, in 1982–2025. The candidate explanatory variables are listed in the table heading. Abbreviations of the climate indices are provided in Table 1. The table presents the coefficient estimates for the intercept and the explanatory variables selected in each model, followed by the model statistics: degrees of freedom (df), Akaike Information Criterion corrected for small sample sizes (AICc), the difference in AICc relative to the best model (delta AICc), and the Akaike weight (weight). The table presents the set of models with a delta AICc < 2. Bold digits indicate variables selected for the best “winter and spring” model. Parameters of this best model are presented in more detail in Table S12; Table S12: Parameters of the best “winter and spring” linear regression models for the dates of the beginning (p10), median (p50), and end (p90) of spring migration of the Eurasian blackcap at Bukowo–Kopań and winter and spring large-scale climate indices selected in the best “winter” and “spring” models, in 1982–2025. The best model had the lowest AICc. The table presents the coefficient estimate (Estimate), its standard error (SE), t-value (t), significance level (p), and Variance Inflation Factor (VIF) for the intercept and the explanatory variables selected by the best model, as well as the Adjusted R^2^ (Adj R^2^) for the entire model; Table S13: Linear regression parameters for the relationships between the dates of the beginning (p10), median (p50), and end (p90) of spring migration of males and females of the Eurasian blackcaps at Bukowo–Kopań in 1982–2025 and the selected large-scale climate indices (Figure 4). The table presents the slope coefficient (β), intercept, standard error (SE), and significance level (p). The slope coefficient (β) estimates the shift in arrival timing (in days) per one-unit increase in the standardised climate index. A negative β value indicates an advance in the date of the analysed percentile with higher values of the climate index; Table S14: Diagnostic tests for the final best “winter and spring” linear regression models for the dates of the beginning (p10), median (p50), and end (p90) of spring migration. The table presents the multiple coefficient of determination (R^2^), adjusted R^2^, (Adj R^2^) the ratio of Adj R^2^ to R^2^, predictive R^2^ (Pred R^2^) calculated based on the Predicted Residual Error Sum of Squares (PRESS) to evaluate model overfitting, and the Durbin–Watson statistic (DW) along with its p-value (DW_p) to assess first-order autocorrelation in the residuals.

## Abbreviations

The following abbreviations are used in this manuscript:

NAO	North Atlantic Oscillation
MOI	Mediterranean Oscillation Index
SCI	Scandinavian Index
IOD	Indian Ocean Dipole
WIN	Winter (December–February)
SPR	Spring (March–May)
