# Supplementary material for: The Influence of Winter and Spring Large-Scale Climate Patterns on the Timing of Male and Female Blackcap Spring Passage at the Baltic Coast
Source: Biology (Basel). 2026 Sep 3;15(17):1518. doi: 10.3390/biology15171518 (PMC13564791; doi:10.3390/biology15171518)
Supplement: Supplementary file 1 [file biology-15-01518-s001.zip › biology-4505939-supplementary.pdf]

The Influence of Winter and Spring Large-Scale Climate Patterns on the Timing of Male and Female Blackcap Spring Passage at the Baltic Coast

Supplementary materials: Figures S1–S3 and Tables S1–S14

Agata Pinszke, Magdalena Remisiewicz *

Bird Migration Research Station, Faculty of Biology, University of Gdańsk,
Wita Stwosza 59, 80-308 Gdańsk, Poland
Correspondence: magdalena.remisiewicz@ug.edu.pl

Figure S1 Long-term trends in large scale climate indices used in the analysis. Statistically significant trends are indicated by an asterisk (*) next to the variable abbreviation.

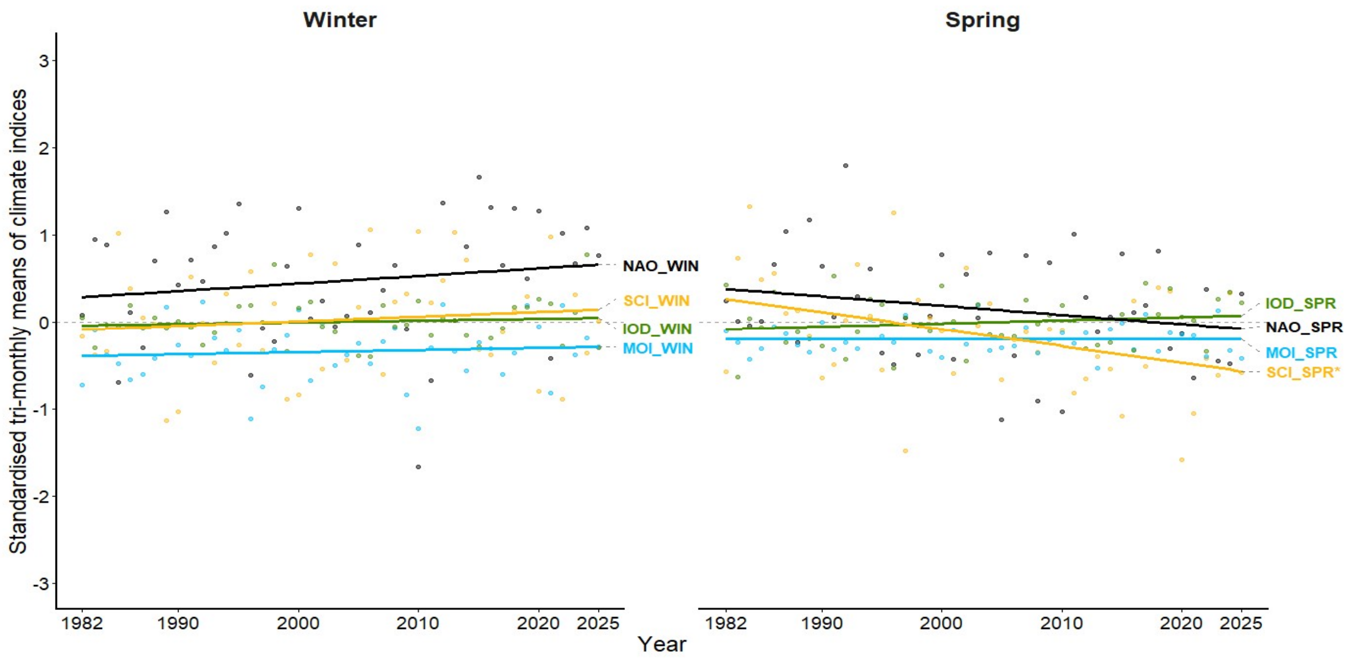

Figure S2. Visual representation of the method of calculating the dates of spring migration phases (p10, p50, and p90) for males and females of Eurasian blackcaps, using the 2009 spring season as an example. The solid stepped lines illustrate the cumulative curve of passage (left y-axis), while the bars show the daily number of captured individuals (right y-axis). The horizontal and vertical lines show how the capture of 10%, 50%, and 90% of all males of females indicated the dates of the respective migration phases. a) Visual representation of deriving the dates of the three phases of passage for males; (b) Visual representation of deriving the dates of the three phases of passage for females.

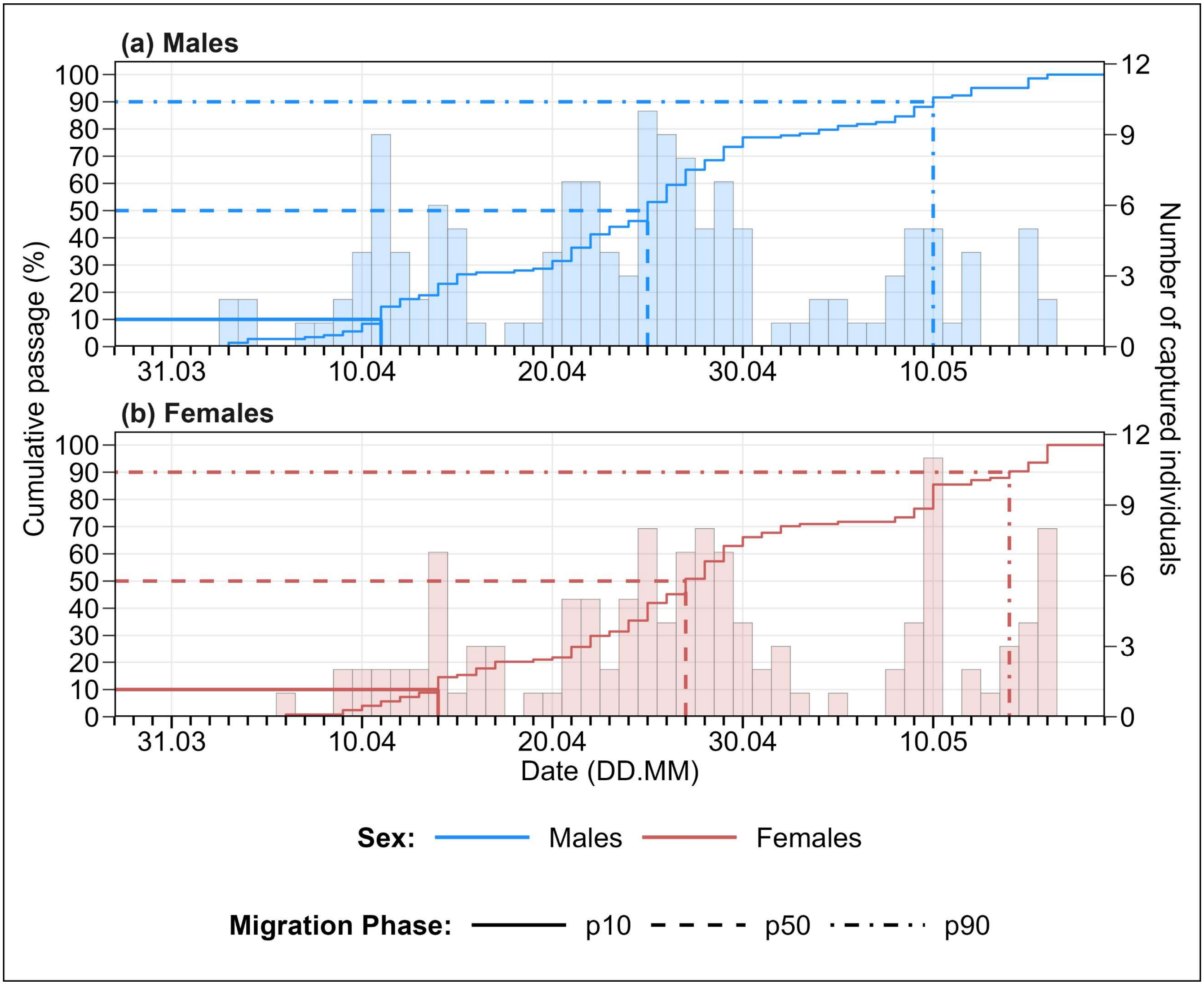

Figure S3. Diagnostic plots for the final best "winter and spring" linear regression models for the dates of the beginning (p10), median (p50), and end (p90) of spring migration. The plots present residuals versus fitted values, normal Q-Q plots, scale-location plots, and residuals versus leverage to assess regression assumptions, including linearity, normality of residuals, and identification of highly influential data points (Cook's distance).

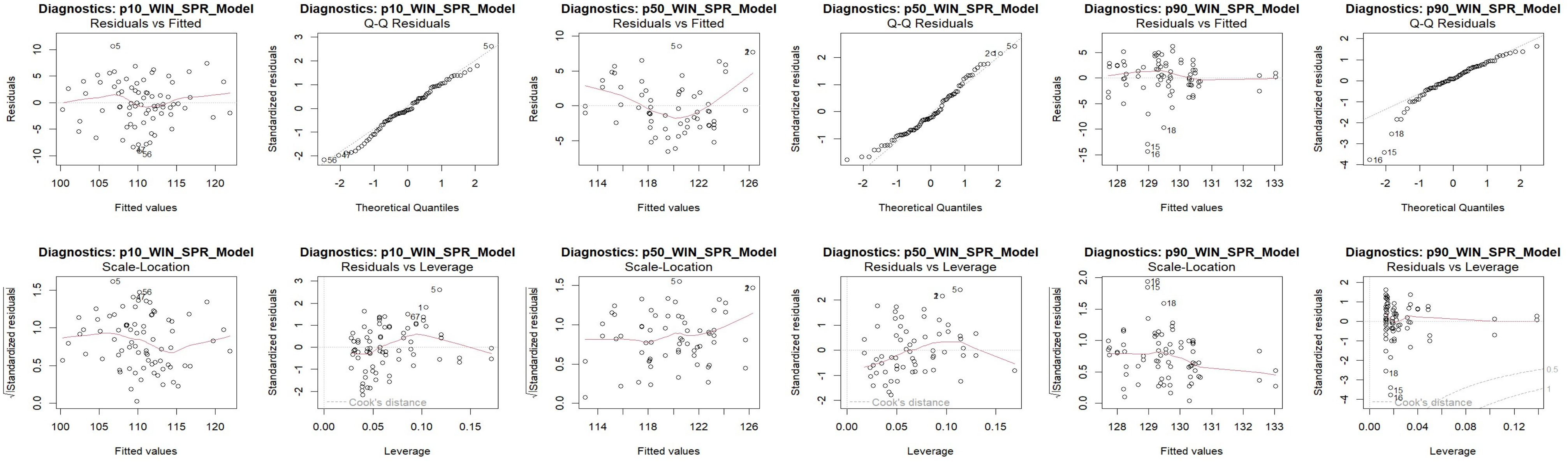

Table S1. Numbers of captured males, females, and all Eurasian blackcaps (*Sylvia atricapilla*) caught, and of the nets used during each spring migration season (26 March–16 May) at Bukowo-Kopań in subsequent years of the period 1982–2025. Grey fields – seasons which were excluded from analyses, because the station did not operate or fewer than 5 females were caught. Total for analyses shows the numbers of individuals considered in analyses.

Year	Females	Males	Total	N nets
1982	44	54	98	57
1983	34	46	80	57
1984	8	13	21	57
1985	5	8	13	42
1986	13	11	24	51
1987	3	5	8	50
1988	15	20	35	45
1989	3	8	11	45
1990	4	4	8	45
1991	6	22	28	45
1992	3	12	15	45
1993	8	11	19	37
1994	23	35	58	47
1995	17	18	35	47
1996	32	32	64	47
1997	47	62	109	47
1998	48	71	119	47
1999	56	77	133	47
2000	38	81	119	47
2001	72	110	182	47
2002	43	91	134	57
2003	54	88	142	51
2004	68	94	162	46
2005	124	154	278	50
2006	133	162	295	50
2007	89	119	208	50
2008	59	91	150	54
2009	124	143	267	50
2010	149	143	292	46
2011	0	0	0	0
2012	105	175	280	35
2013	162	273	435	44
2014	77	140	217	41
2015	73	119	192	48
2016	151	230	381	51
2017	77	126	203	52
2018	123	258	381	52
2019	128	225	353	53
2020	0	0	0	0
2021	156	196	352	46
2022	119	180	299	57
2023	145	165	310	47
2024	148	298	446	48
2025	139	204	343	61
Grand total	2925	4374	7299	
**Total for analyses**	**2912**	**4345**	**7257**	

Table S2. Linear regression parameters for the long-term trends in the dates for the beginning (p10), median (p50), and end (p90) of spring migration of the Eurasian blackcaps at Bukowo-Kopań in 1982–2025 (Figure 3). The table presents the slope coefficient (β), intercept, standard error (SE), and significance level (p). 'β x years' is the estimated total shift in arrival timing (in days) during the analysed period, calculated by multiplying the slope coefficient by the number of years (44 years). A negative β value indicates an advance in the date of the analysed percentile over time.

Percentile	Sex	β	β*44	Intercept	SE	p
p10	Males	-0.208	-9	114.20	0.07	**0.0058**
	Females	-0.197	-9	116.13	0.07	**0.0076**
p50	Males	-0.209	-9	124.55	0.05	**0.0004**
	Females	-0.165	-7	123.78	0.06	**0.0065**
p90	Males	0.001	0	130.05	0.05	0.9899
	Females	0.037	2	128.26	0.06	0.5203

Table S3. Model selection for the linear regression models for the dates of the beginning (p10), median (p50), and end (p90) of spring passage, used as response variables. The explanatory variables in the full models included sex (SexN), standardized year (YearSt), and their interaction (YearSt:SexN). The table presents the coefficient estimates for the intercept and the explanatory variables selected in each model, followed by the model statistics: degrees of freedom (df), Akaike Information Criterion corrected for small sample sizes (AICc), the difference in AICc relative to the best model (delta AICc), and the Akaike weight (weight). The table presents the set of models with a delta AICc < 2. Bold digits indicate variables selected in the best model. Parameters of these best models are presented in more detail in Table S4.

Percentile	(Intercept)	SexN	YearSt	SexN:YearSt	df	AICc	delta AICc	weight
p10	**107.89**	**2.17**	**-2.05**		**4.00**	**477.04**	**0.00**	**0.61**
	108.97		-2.05		3.00	477.96	0.91	0.39
p50	**118.44**		**-1.89**		**3.00**	**439.33**	**0.00**	**1.00**
p90	**129.59**				**2.00**	**428.49**	**0.00**	**0.51**
	130.06	-0.95			3.00	429.55	1.07	0.30
	129.72		0.19		3.00	430.39	1.90	0.20

Table S4. Parameters of the best linear regression models for the dates of the beginning (p10), median (p50), and end (p90) of spring migration used as response variables, and the explanatory variables: standardized year (YearSt), sex (SexN), and their interaction (YearSt:SexN). The best models were selected based on the lowest AICc (Table S4). The table presents the coefficient estimate (Estimate), its standard error (SE), t-value (t), and significance level (p) for the intercept and explanatory variables selected in the best models. The estimates for the YearSt:SexN interaction indicate the change in the degree of protandry over the years.

**Percentile**	**p10**				**p50**				**p90**			
**Variable**	**Estimate**	**SE**	**t**	**p**	**Estimate**	**SE**	**t**	**p**	**Estimate**	**SE**	**t**	**p**
(Intercept)	107.89	0.94	114.38	<0.0001	118.44	0.56	211.23	<0.0001	129.59	0.46	284.42	<0.0001
YearSt	-2.05	0.50	-4.10	0.0001	-1.89	0.39	-4.82	<0.0001				
SexN	2.17	1.24	1.76	0.0833								
YearSt:SexN												

Table S5. Model selection for the “winter” models including NAO (without MOI) of relationships between dates of the beginning (p10), median (p50), and end (p90) of spring migration of the Eurasian blackcap at Bukowo-Kopań and winter large scale climate indices, excluding the winter MOI variable, in 1982–2025. The candidate explanatory variables are listed in the table heading. Abbreviations of the temperature variables are provided in Table 1. The table presents the coefficient estimates for the intercept and the explanatory variables selected in each model, followed by the model statistics: degrees of freedom (df), Akaike Information Criterion corrected for small sample sizes (AICc), the difference in AICc relative to the best model (delta AICc), and the Akaike weight (weight). The table presents the set of models with delta AICc < 2. Bold digits indicate variables selected for the best partial “winter” model. Parameters of this best model are presented in more detail in Table S6.

Percentile	(Intercept)	SexN	YearSt	IOD_WIN	NAO_WIN	SCI_WIN	IOD_WIN:SexN	NAO_WIN:SexN	SCI_WIN:SexN	SexN:YearSt	df	AICc	delta AICc	weight
	**107.97**	**2.17**	**-1.83**		**-1.64**						**5**	**472.52**	**0.00**	**0.46**
p10	107.97	2.17	-1.78	-0.82	-1.84						6	473.51	0.99	0.28
	109.06		-1.83		-1.64						4	473.65	1.14	0.26
p50	**118.50**		**-1.75**		**-1.11**						**4**	**436.63**	**0.00**	**0.72**
	118.49		-1.70		-1.34	-0.39					5	438.47	1.85	0.28
	**129.65**			**1.30**							**3**	**424.34**	**0.00**	**0.49**
p90	130.12	-0.95		1.30							4	425.37	1.03	0.30
	129.63			1.21	-0.34						4	426.05	1.71	0.21

Table S6. Parameters of the best "winter" linear regression models including NAO (without MOI), for the dates of the beginning (p10), median (p50), and end (p90) of spring migration and winter variables in 1982–2025. The best model had the lowest AICc. The table presents the coefficient estimate (Estimate), its standard error (SE), t-value (t), and significance level (p), and Variance Inflation Factor (VIF) for the intercept and the explanatory variables selected by the best model, as well as the Adjusted R² (Adj R²) for the entire model.

**Percentile**	**Variable**	**Estimate**	**SE**	**t**	p	**VIF**	**AdjR²**
p10	(Intercept)	107.97	0.91	118.82	<0.0001	-	25%
	SexN	2.17	1.19	1.82	0.0723	1.00	
	YearSt	-1.83	0.49	-3.76	0.0003	1.03	
	NAO_WIN	-1.64	0.63	-2.60	0.0113	1.03	
p50	(Intercept)	118.50	0.55	216.58	<0.0001	-	27%
	YearSt	-1.75	0.39	-4.50	<0.0001	1.03	
	NAO_WIN	-1.11	0.50	-2.21	0.0301	1.03	
p90	(Intercept)	129.65	0.44	294.26	<0.0001	-	7%
	IOD_WIN	1.30	0.51	2.53	0.0135	-	

Table S7. Model selection for the “winter” models of relationships including MOI (without NAO) between dates of the beginning (p10), median (p50), and end (p90) of spring migration of the Eurasian blackcap at Bukowo-Kopań and winter large scale climate indices, excluding the winter NAO variable, in 1982–2025. The candidate explanatory variables are listed in the table heading. Abbreviations of the temperature variables are provided in Table 1. The table presents the coefficient estimates for the intercept and the explanatory variables selected in each model, followed by the model statistics: degrees of freedom (df), Akaike Information Criterion corrected for small sample sizes (AICc), the difference in AICc relative to the best model (delta AICc), and the Akaike weight (weight). The table presents the set of models with delta AICc < 2. Bold digits indicate variables selected for the best partial “winter” model. Parameters of this best model are presented in more detail in Table S8.

Percentile	(Intercept)	SexN	YearSt	IOD_WIN	MOI_WIN	SCI_WIN	IOD_WIN:SexN	MOI_WIN:SexN	SCI_WIN:SexN	SexN:YearSt	df	AICc	delta AICc	weight
p10	**107.91**	**2.17**	**-1.92**		**-1.09**						**5.00**	**476.43**	**0.00**	**0.20**
	107.96	2.17	-2.09			0.98					5.00	476.87	0.45	0.16
	107.89	2.17	-2.05								4.00	477.04	0.62	0.15
	109.00		-1.92		-1.09						4.00	477.40	0.97	0.13
	109.04		-2.09			0.98					4.00	477.83	1.40	0.10
	108.97		-2.05								3.00	477.96	1.53	0.09
	107.95	2.17	-1.98		-0.77	0.57					6.00	478.19	1.76	0.08
	107.91	2.17	-1.89	-0.50	-1.17						6.00	478.27	1.85	0.08
p50	**118.47**		**-1.78**		**-0.95**						**4.00**	**437.97**	**0.00**	**0.66**
	118.44		-1.89								3.00	439.33	1.36	0.34
p90	**129.65**			**1.30**							**3.00**	**424.34**	**0.00**	**0.47**
	130.12	-0.95		1.30							4.00	425.37	1.03	0.28
	129.61			1.22	-0.47						4.00	425.54	1.20	0.26

Table S8. Parameters of the best "winter" linear regression models including MOI (without NAO), for the dates of the beginning (p10), median (p50), and end (p90) of spring migration at Bukowo-Kopań in 1982–2025 and winter variables in 1982–2025. The best model had the lowest AICc. The table presents the coefficient estimate (Estimate), its standard error (SE), t-value (t), and significance level (p), and Variance Inflation Factor (VIF) for the intercept and the explanatory variables selected by the best model, as well as the Adjusted R^2 (Adj R^2) for the entire model.

Percentile	Variable	Estimate	SE	t	p	VIF	AdjR²
p10	(Intercept)	107.91	0.93	115.80	<0.0001	-	21%
	SexN	2.17	1.22	1.78	0.0797	1.00	
	YearSt	-1.92	0.50	-3.85	0.0003	1.02	
	MOI_WIN	-1.09	0.65	-1.68	0.0979	1.02	
p50	(Intercept)	118.47	0.55	214.79	<0.0001	-	25%
	YearSt	-1.78	0.39	-4.56	<0.0001	1.02	
	MOI_WIN	-0.95	0.51	-1.88	0.0644	1.02	
p90	(Intercept)	129.65	0.44	294.26	<0.0001	-	7%
	IOD_WIN	1.30	0.51	2.53	0.0135	-	

Table S9. Model selection for the “spring models” of relationships between dates of the beginning (p10), median (p50), and end (p90) of spring migration of the Eurasian blackcap at Bukowo-Kopań and spring large scale climate indices in 1982–2025. The candidate explanatory variables, listed in the table heading. Abbreviations of the temperature variables are provided in Table 1. The table presents the coefficient estimates for the intercept and the explanatory variables selected in each model, followed by the model statistics: degrees of freedom (df), Akaike Information Criterion corrected for small sample sizes (AICc), the difference in AICc relative to the best model (delta AICc), and the Akaike weight (weight). The table presents the set of models with a delta AICc < 2. Bold digits indicate variables selected for the best “spring” model. Parameters of this best model are presented in more detail in Table S10.

Percentile	(Intercept)	SexN	YearSt	IOD_SPR	MOI_SPR	NAO_SPR	SCI_SPR	IOD_SPR:SexN	MOI_SPR:SexN	NAO_SPR:SexN	SCI_SPR:SexN	SexN:YearSt	df	AICc	delta	weight
	**107.75**	**2.17**	**-2.90**		**1.13**	**-0.98**	**-2.08**						**7**	**452.89**	**0.00**	**0.35**
	107.71	2.17	-2.87		1.08		-2.16						6	453.66	0.78	0.24
	107.85	2.17	-3.02			-0.93	-2.43						6	454.61	1.72	0.15
	107.79	2.09	-2.90		0.73	-0.98	-2.08		0.79				8	454.78	1.90	0.14
p10	107.68	2.32	-2.90		1.13	-0.98	-1.77				-0.62		8	454.82	1.93	0.13
	**118.46**		**-2.45**			**-1.31**	**-1.27**						**5**	**425.80**	**0.00**	**0.57**
p50	118.44		-2.43	0.56		-1.40	-1.12						6	426.40	0.60	0.43
	**129.59**												**2**	**428.49**	**0.00**	**0.17**
	129.64					-0.57							3	429.29	0.80	0.11
	129.69						-0.44						3	429.30	0.81	0.11
	130.06	-0.95											3	429.55	1.07	0.10
	129.55				0.44								3	429.78	1.30	0.09
	129.58			0.38									3	429.88	1.39	0.09
	129.72		0.19										3	430.39	1.90	0.07
	130.11	-0.95				-0.57							4	430.39	1.91	0.07
	129.73					-0.52	-0.39						4	430.40	1.92	0.07
	130.17	-0.95					-0.44						4	430.41	1.92	0.07
p90	129.63			0.44		-0.63							4	430.46	1.98	0.06

Table S10. Parameters of the best "spring" linear regression models for the dates of the beginning (p10), median (p50), and end (p90) of spring migration at Bukowo-Kopań and spring variables, in 1982–2025. The best model had the lowest AICc. The table presents the coefficient estimate (Estimate), its standard error (SE), t-value (t), and significance level (p), and Variance Inflation Factor (VIF) for the intercept and the explanatory variables selected by the best model, as well as the Adjusted R^2 (Adj R^2) for the entire model.

Percentile	Variable	Estimate	SE	t	p	VIF	AdjR^2
p10	(Intercept)	107.75	0.79	136.99	<0.0001	-	44%
	SexN	2.17	1.03	2.11	0.0383	1.00	
	YearSt	-2.90	0.46	-6.35	<0.0001	1.21	
	MOI_SPR	1.13	0.57	1.98	0.0513	1.14	
	NAO_SPR	-0.98	0.56	-1.74	0.0868	1.01	
	SCI_SPR	-2.08	0.50	-4.18	0.0001	1.36	
p50	(Intercept)	118.46	0.51	234.33	<0.0001	-	37%
	YearSt	-2.45	0.38	-6.37	<0.0001	1.18	
	NAO_SPR	-1.31	0.48	-2.73	0.0079	1.01	
	SCI_SPR	-1.27	0.40	-3.20	0.0021	1.19	
p90	(Intercept)	129.59	0.46	284.42	<0.0001	-	0%

Table S11. Model selection for the “winter and spring” models of relationships between dates of the beginning (p10), median (p50), and end (p90) of spring migration of the Eurasian blackcap at Bukowo-Kopań and for combined winter and spring temperature variables, in 1982–2025. The candidate explanatory variables are listed in the table heading. Abbreviations of the climate indices are provided in Table 1. The table presents the coefficient estimates for the intercept and the explanatory variables selected in each model, followed by the model statistics: degrees of freedom (df), Akaike Information Criterion corrected for small sample sizes (AICc), the difference in AICc relative to the best model (delta AICc), and the Akaike weight (weight). The table presents the set of models with a delta AICc < 2. Bold digits indicate variables selected for the best “winter and spring” model. Parameters of this best model are presented in more detail in Table S12.

Percentile	(Intercept)	SexN	YearSt	MOI_SPR	NAO_SPR	NAO_WIN	SCI_SPR	IOD_WIN	df	logLik	AICc	delta AICc	weight
p10	**107.89**	**2.17**	**-2.79**			**-1.75**	**-2.55**		**6**	**-216.61**	**446.43**	**0.00**	**0.55**
	107.81	2.17	-2.72	0.76		-1.61	-2.31		7	-215.60	446.85	0.42	0.45
p50	**118.49**		**-2.34**		**-1.00**	**-0.81**	**-1.31**		**6**	**-206.06**	**425.34**	**0.00**	**0.45**
	118.46		-2.45		-1.31		-1.27		5	-207.47	425.80	0.46	0.36
	118.46		-2.27			-1.17	-1.39		5	-208.06	426.98	1.64	0.20
p90	**129.65**							**1.30**	**3**	**-209.00**	**424.34**	**0.00**	**1.00**

Table S12. Parameters of the best "winter and spring" linear regression models for the dates of the beginning (p10), median (p50), and end (p90) of spring migration of the Eurasian blackcap at Bukowo-Kopań and winter and spring large scale climate indices selected in the best "winter" and "spring" models, in 1982–2025. The best model had the lowest AICc. The table presents the coefficient estimate (Estimate), its standard error (SE), t-value (t), significance level (p), and Variance Inflation Factor (VIF) for the intercept and the explanatory variables selected by the best model, as well as the Adjusted R^2 (Adj R^2) for the entire model.

Percentile	Variable	Estimate	SE	t	p	VIF	AdjR²
q10	(Intercept)	107.89	0.76	142.15	<0.0001	-	48%
	NAO_WIN	-1.75	0.53	-3.31	0.0015	1.03	
	SCI_SPR	-2.55	0.45	-5.68	<0.0001	1.18	
	SexN	2.17	0.99	2.18	0.0323	1.00	
	YearSt	-2.79	0.44	-6.33	<0.0001	1.21	
q50	(Intercept)	118.49	0.50	236.99	<0.0001	-	39%
	NAO_SPR	-1.00	0.51	-1.96	0.0541	1.17	
	NAO_WIN	-0.81	0.50	-1.64	0.1057	1.20	
	SCI_SPR	-1.31	0.39	-3.34	0.0013	1.20	
	YearSt	-2.34	0.39	-6.07	<0.0001	1.22	
q90	(Intercept)	129.65	0.44	294.26	<0.0001	-	7%
	IOD_WIN	1.30	0.51	2.53	0.0135	-	

Table S13. Linear regression parameters for the relationships between the dates of the beginning (p10), median (p50), and end (p90) of spring migration of males and females of the Eurasian blackcaps at Bukowo-Kopań in 1982–2025 and the selected large-scale climate indices (Figure 4). The table presents the slope coefficient (β), intercept, standard error (SE), and significance level (p). The slope coefficient (β) estimates the shift in arrival timing (in days) per one-unit increase in the standardised climate index. A negative β value indicates an advance in the date of the analysed percentile with higher values of the climate index.

Percentile	Climate index	Sex	β	Intercept	SE	p
p10	NAO_WIN	Males	-1.99	109.26	0.98	**0.050**
		Females	-2.11	111.43	0.95	**0.033**
	SCI_SPR	Males	-0.98	109.58	0.80	0.230
		Females	-1.60	111.90	0.76	**0.042**
p50	NAO_WIN	Males	-1.64	119.60	0.79	**0.047**
		Females	-1.36	119.86	0.80	0.098
	NAO_SPR	Males	-1.32	119.79	0.84	0.125
		Females	-1.15	120.02	0.84	0.180
	SCI_SPR	Males	0.02	119.67	0.67	0.982
		Females	-0.79	120.11	0.65	0.227
p90	IOD_WIN	Males	1.32	130.12	0.65	0.051
		Females	1.28	129.17	0.80	0.119

Table S14. Diagnostic tests for the final best "winter and spring" linear regression models for the dates of the beginning (p10), median (p50), and end (p90) of spring migration. The table presents the multiple coefficient of determination (R^2), adjusted R^2, (Adj R^2) the ratio of Adj R^2 to R^2, predictive R^2 (Pred R^2) calculated based on the Predicted Residual Error Sum of Squares (PRESS) to evaluate model overfitting, and the Durbin-Watson statistics (DW) along with its p-value (DW_p) to assess first-order autocorrelation in the residuals.

Percentile	R^2	Adj R^2	Adj R^2/R^2	Pred R^2	PRESS	DW	DW_p
p10	0.506	0.478	0.945	0.433	1525.44	1.069	0.000
p50	0.422	0.389	0.923	0.337	1155.22	0.913	0.000
p90	0.08	0.067	0.844	0.043	1132.46	1.250	0.000
